# Supplementary material for: Identification of Candidate Genes for Rind Color and Bloom Formation in Watermelon Fruits Based on a Quantitative Trait Locus-Seq
Source: Plants (Basel). 2022 Oct 17;11(20):2739. doi: 10.3390/plants11202739 (PMC9611755; doi:10.3390/plants11202739)
Supplement: Supplementary file 1 [file plants-11-02739-s001.zip › plants-1945771-supplementary/Table S3.pdf]

Table S3. Statistics of sequencing data of raw read and genome coverage of trimmed read after equalizing.

DNA samples	No. of F_2 plants in bulk	No. of reads	Average length(bp)	Total length(bp)	Q30(%)[z]	Coverage[y]	Coverage[y] after equalizing
SIT55616RN	-	23,238,593	151	3,509,027,543	93.57	≒16.51X	
		23,238,593		3,509,027,543			
FD061129	-	22,901,520	151	3,458,129,520	93.28	≒16.27X	
		22,901,520		3,458,129,520			
DG-bulk	40	64,221,090	151	9,697,384,590	94.83	≒45.63X	≒34.54X
		64,221,090		9,697,384,590			
LG-bulk	9	62,602,038	151	9,452,907,738	94.73	≒44.48X	≒34.54X
		62,602,038		9,452,907,738			
B-bulk	40	62,733,048	151	9,472,690,248	94.35	≒44.85X	≒33.86X
		62,733,048		9,472,690,248			
Bl-bulk	40	63,529,167	151	9,592,904,217	94.5	≒45.14X	≒33.86X
		63,529,167		9,592,904,217			

[z] Ratio of bases that have quality score of over 30.

[y] Total raw read length of each sample divided by estimated genome size (425 Mb).
