# Supplementary material for: Identification of Candidate Genes for Rind Color and Bloom Formation in Watermelon Fruits Based on a Quantitative Trait Locus-Seq
Source: Plants (Basel). 2022 Oct 17;11(20):2739. doi: 10.3390/plants11202739 (PMC9611755; doi:10.3390/plants11202739)
Supplement: Supplementary file 1 [file plants-11-02739-s001.zip › plants-1945771-supplementary/Table S4.pdf]

Table S4. Statistics of Single nucleotide polymorphism calling for bulked DNA samples.

Chromosome.	No. of SNPs for rind color			No. of SNPs for bloom		
	DG-bulk	LG-bulk	comparable	B-bulk	Bl-bulk	comparable
1	6,968	7,129	7,043	7,132	6,770	7,103
2	16,576	15,805	16,843	16,823	16,382	17,053
3	8,745	7,283	8,564	8,483	8,664	8,727
4	12,904	12,534	13,163	13,123	12,989	13,451
5	25,438	24,781	25,806	25,690	25,223	25,981
6	13,029	8,864	12,975	12,823	13,082	13,275
7	19,911	20,079	20,432	20,412	20,175	20,535
8	9,239	7,902	9,084	8,859	9,153	9,183
9	27,646	25,043	27,529	27,036	27,646	27,766
10	7,322	7,197	7,118	7,184	7,213	7,130
11	8,271	7,606	8,267	8,568	8,376	8,558
Scaffolds(102ea)	870	938	932	922	884	930
Total	156,919	145,161	157,756	157,055	156,557	159,692
